# Representation of conspecific vocalizations in amygdala of awake marmosets

**DOI:** 10.1093/nsr/nwad194

**Published:** 2023-07-13

**Authors:** Guoqiang Jia, Siyi Bai, Yingxu Lin, Xiaohui Wang, Lin Zhu, Chenfei Lyu, Guanglong Sun, Kang An, Anna Wang Roe, Xinjian Li, Lixia Gao

**Affiliations:** Department of Neurology of the Second Affiliated Hospital and Interdisciplinary Institute of Neuroscience and Technology, Zhejiang University School of Medicine, Hangzhou 310029, China; Department of Neurology of the Second Affiliated Hospital and Interdisciplinary Institute of Neuroscience and Technology, Zhejiang University School of Medicine, Hangzhou 310029, China; Key Laboratory of Biomedical Engineering of Ministry of Education, College of Biomedical Engineering and Instrument Science, Zhejiang University, Hangzhou 310027, China; Department of Neurology of the Second Affiliated Hospital and Interdisciplinary Institute of Neuroscience and Technology, Zhejiang University School of Medicine, Hangzhou 310029, China; Key Laboratory of Biomedical Engineering of Ministry of Education, College of Biomedical Engineering and Instrument Science, Zhejiang University, Hangzhou 310027, China; Department of Neurology of the Second Affiliated Hospital and Interdisciplinary Institute of Neuroscience and Technology, Zhejiang University School of Medicine, Hangzhou 310029, China; Key Laboratory of Biomedical Engineering of Ministry of Education, College of Biomedical Engineering and Instrument Science, Zhejiang University, Hangzhou 310027, China; Department of Neurology of the Second Affiliated Hospital and Interdisciplinary Institute of Neuroscience and Technology, Zhejiang University School of Medicine, Hangzhou 310029, China; Department of Neurology of the Second Affiliated Hospital and Interdisciplinary Institute of Neuroscience and Technology, Zhejiang University School of Medicine, Hangzhou 310029, China; Department of Neurology of the Second Affiliated Hospital and Interdisciplinary Institute of Neuroscience and Technology, Zhejiang University School of Medicine, Hangzhou 310029, China; College of Information, Mechanical and Electrical Engineering, Shanghai Normal University, Shanghai 201418, China; Department of Neurology of the Second Affiliated Hospital and Interdisciplinary Institute of Neuroscience and Technology, Zhejiang University School of Medicine, Hangzhou 310029, China; MOE Frontier Science Center for Brain Science and Brain-Machine Integration, School of Brain Science and Brain Medicine, Zhejiang University, Hangzhou 310058, China; Key Laboratory of Biomedical Engineering of Ministry of Education, College of Biomedical Engineering and Instrument Science, Zhejiang University, Hangzhou 310027, China; Department of Neurology of the Second Affiliated Hospital and Interdisciplinary Institute of Neuroscience and Technology, Zhejiang University School of Medicine, Hangzhou 310029, China; MOE Frontier Science Center for Brain Science and Brain-Machine Integration, School of Brain Science and Brain Medicine, Zhejiang University, Hangzhou 310058, China; Key Laboratory of Medical Neurobiology of Zhejiang Province, Zhejiang University School of Medicine, Hangzhou 310020, China; Department of Neurology of the Second Affiliated Hospital and Interdisciplinary Institute of Neuroscience and Technology, Zhejiang University School of Medicine, Hangzhou 310029, China; MOE Frontier Science Center for Brain Science and Brain-Machine Integration, School of Brain Science and Brain Medicine, Zhejiang University, Hangzhou 310058, China; Key Laboratory of Biomedical Engineering of Ministry of Education, College of Biomedical Engineering and Instrument Science, Zhejiang University, Hangzhou 310027, China

**Keywords:** vocalization, non-human primate, marmoset, amygdala

## Abstract

Human speech and animal vocalizations are important for social communication and animal survival. Neurons in the auditory pathway are responsive to a range of sounds, from elementary sound features to complex acoustic sounds. For social communication, responses to distinct patterns of vocalization are usually highly specific to an individual conspecific call, in some species. This includes the specificity of sound patterns and embedded biological information. We conducted single-unit recordings in the amygdala of awake marmosets and presented calls used in marmoset communication, calls of other species and calls from specific marmoset individuals. We found that some neurons (47/262) in the amygdala distinguished ‘Phee’ calls from vocalizations of other animals and other types of marmoset vocalizations. Interestingly, a subset of Phee-responsive neurons (22/47) also exhibited selectivity to one out of the three Phees from two different ‘caller’ marmosets. Our findings suggest that, while it has traditionally been considered the key structure in the limbic system, the amygdala also represents a critical stage of socially relevant auditory perceptual processing.

## Teaser

This study reveals a crucial role of the amygdala in processing the conspecific vocalization.

## INTRODUCTION

Conspecific vocalization (CV) is important for social communication in non-human primates [1] and has been proposed to serve as a prototype for human language [2–4]. The calls of non-human primates are acoustically complex sounds with unique spectral and temporal components [5] and are thought to carry distinct behavioral meanings within species [1,6]. Electrophysiological studies have shown that the primary auditory cortex (A1) is responsive to the spectral and temporal aspects of these calls [5,7] as well as to natural calls themselves [5,7–9]. As suggested by neuroimaging studies, these responses exist in a gradient of representation along the superior temporal lobe (ST), in which the caudal region is preferentially activated by acoustic features and the rostral region is dominated by responses to integrated conspecific calls [3,9–12]. These findings suggest a functional hierarchy from general acoustic features to more specific communication-related signals. However, what has not been incorporated into this view is the stimulus specificity of different calls, one which specifies not only the conspecifics but also the embedded biological meaning, and emotional and environmental information. Behaviorally, recognition of specific calls is crucial for social interaction [13–16], which may rely on the limbic system. Here, we hypothesize that another important brain center in the communication hierarchy lies in the amygdala, a primary center for limbic processing.

In non-human primates, the amygdala is known to receive inputs from thalamic [17] and cortical auditory pathways [18]. It also projects to prefrontal areas [19] which process and integrate conspecific calls in the context of factors such as familiarity, motivation and social signal [20]. Thus, the anatomy indicates it is a key limbic link between sensory inputs and cognitive responses. This hypothesis is further supported by studies that have shown that the amygdala is activated by non-linguistic emotional vocalizations [21] and is a locus for representing the valence of emotion in both auditory and visual modalities [22,23]. Several studies in rodents and bats proved that the neurons in the amygdala could be activated by conspecific calls [24–31]. In addition, different animals, especially non-human primates, have different abilities to modulate their vocalization [32]. However, it remains elusive how the amygdala of marmosets encodes conspecific calls. In the current study, we recorded single units in the amygdala of awake marmosets presented with different acoustic sound patterns, including natural sounds, artificial sounds, other species’ calls, conspecific calls and three Phee calls from two callers. We report the presence of individual neurons in the amygdala that can distinguish different call types, while having low sensitivity to basic acoustic features, which indicates higher or secondary auditory processing in vocal perception. This finding significantly strengthens the view of the amygdala as a key player in the social communication hierarchy.

## RESULTS

### Representation of conspecific vocalizations in the amygdala

**Figure 1.**
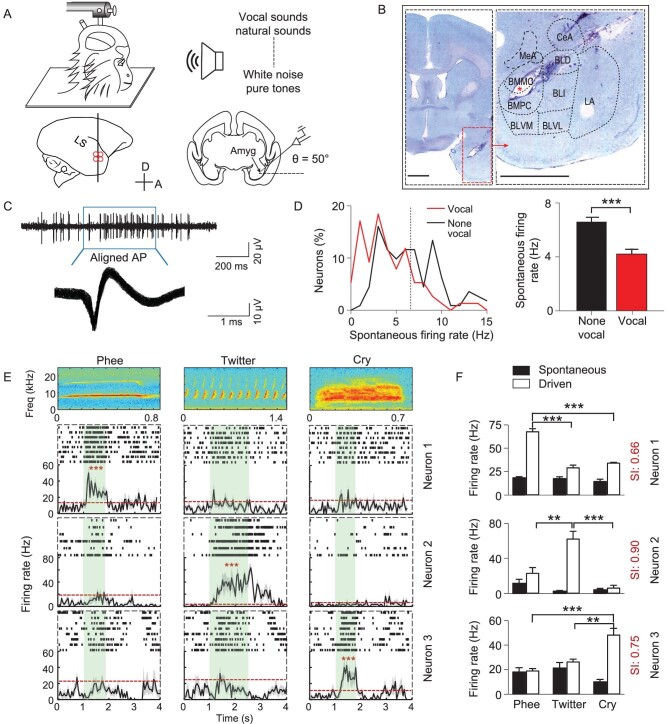
Selective responses of amygdala neurons to specific marmoset calls. (A) Sketch of the animal preparation and single-unit recording procedure in the amygdala of an awake head-fixed marmoset. (B) Images of a Nissl-stained section containing an electrolytic lesion in the amygdala (Amyg) labeled by an asterisk. Left, overall view of a coronal section. Right, magnified view of the amygdala. Scale bar, 2 mm. *, lesion site. (C) A sampled raw trace of single-unit recording (top) and spike waveforms of the same unit (bottom). (D) Left, the distribution of the spontaneous firing rates of call-responsive (vocal) and non-responsive (non-vocal) neurons; right, the averaged spontaneous firing rates of call-responsive and non-responsive neurons (in black). The dashed vertical lines on the left indicate the averaged firing rate. (E) Three examples of raster and trial-averaged firing rates (6–8 trials) of Phee, Twitter and Cry neurons (Student's paired t-test). Top, spectrograms of Phee, Twitter and Cry calls. Horizontal dashed line, spontaneous firing rate averaged over the period before the onset of vocal stimuli across all trials. Green shading, periods of acoustic stimuli. (F) Spontaneous and driven rates averaged throughout vocal stimuli (6–8 repetitions) for the three example neurons shown in (E) (two-way ANOVA followed by Student's t-test, F = 61.23, 16.55,11.22, *P* < 0.001). SI, selectivity index.

**Figure 2.**
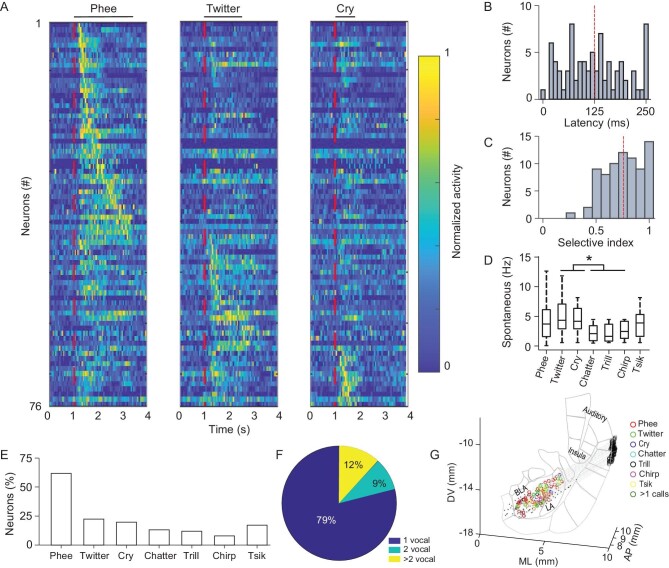
Conspecific vocalization representation in the amygdala. (A) Population activation of 76 amygdala neurons call-responsive for Phee (left), Twitter (middle) and Cry (right) calls. Red dashed lines, onset of call stimuli. (B) Distribution of response latency of 76 amygdala call-responsive neurons. Red line, average latency of all neurons. (C) Distribution of SI of call-responsive neurons. Red line, average SI of 76 neurons. (D) The averaged spontaneous firing rate of seven call-responsive neurons. (E) Proportions of amygdala call-responsive neurons in response to seven calls. (F) Proportions of amygdala call-responsive neurons in response to one, two and more than two calls. (G) Reconstructed recording map of call-responsive neurons in the marmoset amygdala. Gray dots, no response to vocalization. Red, Phee; green, Twitter; blue, Cry; cyan, Chatter; black, Trill; magenta, Chirp; yellow, Tsik; light brown, response to >1 call.

To characterize neural activity in the amygdala, we used a tungsten electrode to reach the amygdala from a 1 mm craniotomy inside a laterally placed recording chamber at 50° relative to the horizontal plane (Fig. 1A–C). Meanwhile, seven CVs including Phee, Twitter, Cry, Chatter, Trill, Chirp and Tsik (Fig. S1A, supplementary Tables 1–2) that had been recorded from other marmosets (callers) from the same colony (see Methods) were played back to animals (listeners). Interestingly, single amygdala neurons responded specifically to a single marmoset call exemplar among seven tested calls, i.e. the activity evoked by one specific marmoset call was several-fold stronger than that evoked by other CVs (Fig. 1E, F, Fig. S1B). Different neurons in the amygdala were activated by different calls (Fig. 1E, F, Fig. S1B). In this study, if the driven rate of a neuron to any one of the marmoset calls is significantly higher than the two standard deviations of the averaged spontaneous rate (mean + 2*std, paired test, *P* < 0.05), the neuron is defined as a call-responsive neuron; otherwise, the neuron is defined as a call-non-responsive neuron. In addition, we calculated the spontaneous firing rate of the amygdalar neurons and found that the spontaneous firing rate of call-responsive neurons was less than non-responsive neurons (Fig. 1D). In total, 76 out of 262 neurons were activated by marmoset calls and displayed diverse temporal firing patterns and varying response durations (Fig. 2A). In contrast to A1 neurons [33], the amygdala call-responsive neurons showed a longer response latency (Fig. 2B) and a higher selectivity index to CVs (Fig. 2C). The neurons exhibited higher spontaneous firing rates in response to Phee, Twitter and Cry than those responding to Chatter, Trill and Chirp (Fig. 2D). And the spontaneous firing rate is not correlated with the selectivity index (Fig. S1C). Furthermore, 62% of call-responsive neurons in the amygdala are Phee-responsive neurons (Fig. 2E, S2A and B), distinct from A1, which had a higher proportion of Twitter-responsive neurons [8]. Intriguingly, most of the call-responsive neurons in the amygdala were only activated by one specific call (Fig. 2F, S2C and D). To further identify the location of call-responsive neurons, a recording map was reconstructed based on the depth of the electrode and anatomical results (Fig. 1B). We found that the call-responsive neurons were distributed in the basal lateral and lateral regions of the amygdala. Most neurons in the dorsal and middle basal lateral amygdala (BLA) were Phee-responsive (Fig. 2G).

### High selectivity of marmoset amygdala neurons to conspecific calls

**Figure 3.**
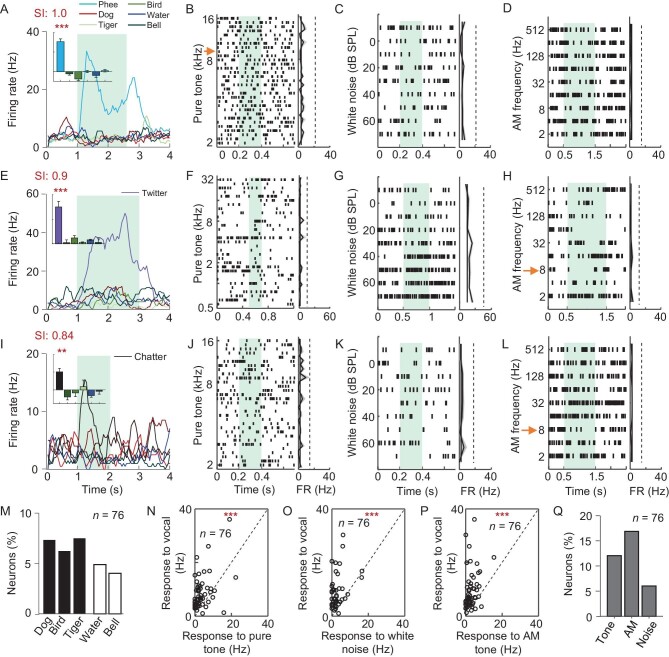
High selectivity of amygdala neurons to conspecific calls in the marmoset. (A) Example of a Phee-specific neuron in response to calls from other species, water and bell sound. Top left, average responses to different acoustic stimuli (one-way ANOVA F = 48.48, *P* < 0.001). (B–D) Left, Raster plot (summation of 6–8 trials) of a Phee neuron in response to (B) pure tones at varying frequencies, (C) broad-band white noise at different sound levels and (D) sinusoidal amplitude modulated (sAM) tones at varying modulation frequencies. Right, tuning curve of the Phee-responsive neuron to different conventionally structured sounds. Red arrow in (B), the fundamental frequency (10 kHz) of the Phee call. (E) Example of a Twitter neuron (one-way ANOVA, F = 14.32, *P* < 0.001), and its response to (F) pure tones, (G) white broad-band noise and (H) amplitude-modulated tones. Red arrow in (H), modulation frequency similar to the Twitter call (8 Hz). (I) Example of a Chatter neuron (one-way ANOVA, F = 2.47, *P* < 0.01), and its response to (J) pure tones, (K) broad-band white noise and (L) amplitude-modulated tones. Red arrow, modulation frequency similar to the Chatter call (8 Hz). (M) Percentages of call-responsive neurons showing significant spiking response to dog, bird, tiger, water and bell. (N–P) Firing rates of call-responsive neurons in the amygdala in response to CVs, plotted against those in response to (N) pure tones, (O) broad-band white noise and (P) sAM tones (paired t-test, *P* < 0.001). (Q) Percentages of call-responsive neurons showing a significant response to pure tones, sAM tones and broad-band white noise.

First, to further identify the specificity of conspecific call representation in the amygdala, calls from other species (dog, tiger and bird, Fig. S3A) were delivered. Our results showed that amygdala neurons showed lower activation to these stimuli in contrast to robust activation by specific marmoset calls (Fig. 3A, E and I). Second, natural sound (water flow) and artificial tone-like acoustic stimulation (bell) were delivered and only a few neurons exhibited evoked response (Fig. 3A, E and I). Quantitatively, calls from other species, natural sound and artificial sound activated around 5%–8% of amygdala call-responsive neurons (Fig. 3M, Fig. S3B and C). Thus, amygdala neurons prefer conspecific calls to other acoustic stimuli.

A hypothesis of conspecific call-responsive neurons in the amygdala is based on the temporal and spectral information embedded in conspecific calls. To test this possibility, pure-tone (Fig. 3B, F and J), white broad-band noise (Fig. 3C, G and K) and sinusoidal amplitude-modulated (sAM) tones (Fig. 3D, H and L) were played to animals. First, the spectral and temporal structure of the Phee call is close to a pure tone with a dominant frequency centered at 7–10 kHz (Fig. S1A). Interestingly, the amygdala neuron that responds to a Phee call (Fig. 3A) was not activated by pure tones with frequencies varying from 7 to 10 kHz (Fig. 3B and Fig. S4A). Moreover, white broad-band noise at different sound pressure levels (Fig. 3C and Fig. S4B) and amplitude-modulated (AM) tones at different modulation frequencies (Fig. 3D and S4C) did not activate amygdala neurons either. Second, Twitter and Chatter calls have salient temporal structures (Fig. S1A) embedded with 7–8 Hz AM stimuli. Similarly, both amygdala neurons, in response to a Twitter call (Fig. 3E) and Chatter (Fig. 3I), were not driven by 7–8 Hz sAM tones (Fig. 3H and L) or white noise at similar sound pressure levels (60 dB; Fig. 3G and K). Statistically, the amygdala call-responsive neurons exhibited significantly higher activity in response to conspecific calls than pure tones (Fig. 3N), broad-band white noise (Fig. 3O) and sAM tones (Fig. 3P). Moreover, <20% (76 neurons in total) of call-responsive neurons were activated by laboratory-composed acoustic stimuli (Fig. 3Q, S3D and E). Thus, most amygdala neurons selectively encode aspects of marmoset calls beyond the basic acoustic features.

### Heterogeneous activation of amygdala neurons by time-reversed calls

**Figure 4.**
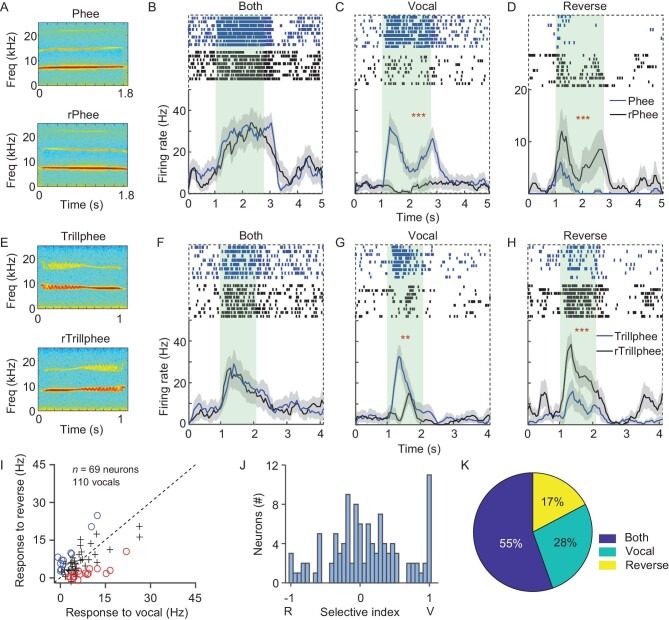
Heterogeneous activation by time-reversed calls. (A) Spectrograms of Phee and time-reversed Phee (rPhee). (B–D) Three example neurons showing responses to (C) Phee (blue), (D) rPhee (black) and (B) both (Student's t-test, C, D, *P* < 0.001). (E) Spectrograms of Trillphee and time-reversed Trillphee (rTrillphee). (F–H) Three example neurons showing responses to (G) Trillphee (blue), (H) rTrillphee (black) and (F) both (Student's t-test, G, *P* < 0.01, H, *P* < 0.001). (I) Firing rates of amygdala neurons in response to natural CVs, plotted against those in response to time-reversed CVs. (J) Selectivity index of 69 amygdala neurons in response to 110 vocal stimuli. (K) Percentages of call-responsive neurons responding to natural calls (vocal), time-reversed calls (reverse) and both.

To further explore the importance of CV representation in the amygdala, we played both natural and time-reversed marmoset calls (Fig. 4A and E) that had the same spectral content and similar acoustic complexity as natural calls but with switched temporal sequence (supplementary Table 3) [33]. Interestingly, in contrast to the A1 neurons exhibiting stronger responses to natural calls than to time-reversed versions [5,33], more than half of the CV-responsive neurons in the amygdala displayed similar firing rates and temporal patterns to both natural and time-reversed calls (Fig. 4B, F, I, J and K). While some neurons exhibited equivalent responses to natural and time-reversed calls, some call-responsive neurons showed preference for natural calls (Fig. 4C, G and I) and others for time-reversed calls (Fig. 4D, H and I). In total, 55% (39 out of 69) of amygdala neurons displayed similar activation by natural and time-reversed calls; 28% preferred natural calls, and 17% preferred time-reversed calls (Fig. 4K). In addition, we also compared the neural activity of a time-reversed disyllabic complex call: Trillphee. We found that 36% of Trillphee responsive neurons responded to both natural and time-reversed Trillphee, which was much lower than the percentage of monosyllabic responsive neurons. 17% of Trillphee responsive neurons responded solely to natural calls and 45% only responded to time-reversed Trillphee. The higher proportion of time-reversed Trillphee-responsive neurons may be due to their preference for the Phee segment of Trillphee which is at the beginning of time-reversed Trillphee (Fig. S5). Therefore, amygdala neurons displayed heterogeneous activation to time-reversed calls compared to natural calls.

### Amygdala neurons respond differently to different Phee calls

Phee is the most commonly used marmoset call for vocal communications, and can be used in different behavioral contexts such as territorial marking in the home cage, reuniting the group [34], and in the natural environment. Our results showed that 62% of call-responsive neurons in the amygdala were Phee-selective and exhibited a significant response to the Phee exemplar but not to any other call exemplars (Fig. 2D). We next investigated whether different Phee calls evoke similar responses because of their similar spectral content and temporal structure. To address this question, three Phee calls from two marmosets (Fig. S6A) were recorded and tested on 47 Phee-responsive neurons in the amygdala. We first analyzed the spectrotemporal features of Phee calls from animals M and X. The Phee call from animal M has a shorter duration, larger entropy, broader frequency bandwidth and lower end-frequency than the Phee call of animal X. Less difference was found in the spectrotemporal features between the two Phee calls produced by animal X (supplementary Table 4). Then we compared the neuronal activity of amygdala neurons responding to different Phees; interestingly, we found that most Phee-responsive neurons were activated by one or two Phees (Fig. S6B, C, E, F and G). These results revealed that Phee-responsive neurons in the amygdala can differentiate between Phee calls, similar to previous reports that A1 neurons exhibit distinct responses to different Twitter calls [5]. However, the Phee-responsive index was smaller than that of seven different CVs (Fig. 2C, Fig. S6H). Furthermore, we found, strikingly, that 47% of Phee-responsive neurons were activated only by Phee calls from animal M (Fig. S5E) or from animal X (Fig. S6F and I). Thus, amygdala neurons respond distinctly to different Phee calls and this further indicates that amygdala call-responsive neurons have stimulus specificity.

### Phee integrity is essential for the activation of amygdala Phee-responsive neurons

**Figure 5.**
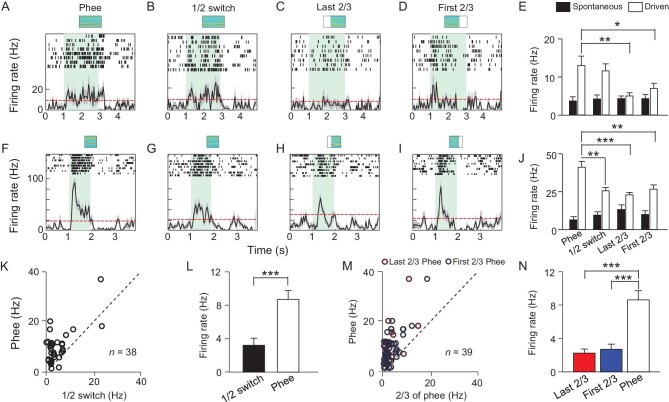
Phee integrity is essential for the activation of amygdala Phee-responsive neurons. (A–D) The response of one example neuron to (A) Phee, (B) time-switched Phee and (C and D) partially removed Phee calls. (E) Statistical analysis of the responses of an example neuron (two-way ANOVA, F = 6.82, *P* < 0.001, with Student t-test). (F–I) Neural responses of another example neuron. (J) Statistical analysis of the second neuron (two-way ANOVA, F = 22.9, *P* < 0.001, with Student t-test). (K) Firing rates of Phee calls, plotted against those of time-switched Phee calls. (L) Average response of Phee-responsive neurons to Phee calls and time-switched Phee calls (paired t-test, *P* < 0.001). (M) Firing rates of Phee calls plotted against those of partially removed Phee calls. (N) Average response of Phee-responsive neurons to Phee calls and partially removed Phee calls (one-way ANOVA F = 20.33, *P* < 0.001).

Our results above demonstrate that some neurons in the amygdala can distinguish Phee calls from other marmoset calls and even distinguish different Phee calls with similar acoustic features. However, we wondered whether this distinct response of Phee-responsive neurons depends on the integrity of the Phees. To address this question, we first cut the Phee calls into halves from the middle and switched the sequence of paired segments (Fig. 5B and G, top, supplementary Table 5 for acoustic features), which did not change the energy power and spectrogram of the Phee calls. We found that the firing rate of Phee-responsive neurons in response to switched Phee was significantly lower than the response to the original Phee stimulation (Fig. 5F, G and J–L). Next, we created partially removed Phee calls (first two-thirds or last two-thirds of Phee calls, supplementary Table 5) and examined the neural activity of amygdala neurons (Fig. 5A, C, D, F, H and I). This modification shortened the time and reduced the spectrum structure of the Phee calls. We found that Phee-responsive neurons exhibited decreased responses to partially removed Phee calls compared with original Phee calls (Fig. 5E, H–J, M and N). These results demonstrate that the integrity of Phee calls is necessary for the activation of amygdala Phee-responsive neurons.

## DISCUSSION

How biologically meaningful and socially relevant objects are perceived as being is one of the pivotal issues in visual and auditory neuroscience. The primary visual cortex mainly processes and integrates basic visual elements, such as orientation, motion, contrast, spatial frequency and ocular dominance [35–37]. These elements are integrated into natural objects such as ‘in face patches’ that are processed and perceived in several discrete cortical regions such as the V4, inferior temporal lobe, superior temporal lobe [38,39] and prefrontal cortex [40,41]. Analogous to the visual system, A1 is crucial for processing spectral and temporal sound components [42,43], although part of A1 and related cortical regions in the temporal lobe prefer acoustically complex sounds, such as conspecific calls [5,33,44]. Most neurons in the A1 that prefer conspecific calls can also be driven by spectrally and temporally similar stimuli and are spatially mixed with spectral- and temporal-sound-driven neurons [5,33]. Thus, it remains unclear how the integrated information of CVs is processed. The amygdala receives afferent inputs from the auditory thalamus [17] and is reciprocally connected with the auditory cortex [18]. Meanwhile, electrical stimulation in the amygdala can trigger different calls in cat and squirrel monkeys via different pathways [45] including the periaqueductal region [46]. In humans and macaque monkeys, the amygdala was activated when non-language emotional sounds [21] or complex social stimuli [47] were presented. Moreover, the amygdala of non-human primates processes and integrates emotional information both in the auditory and visual modalities [22,23], suggesting that it may be involved in processing conspecific calls.

fMRI studies in both human and non-human primates have demonstrated that some neurons in the temporal lobe, including A1, display stronger activation by conspecific calls than calls from other species [3,4,9,11]. Furthermore, conspecific calls evoke greater activation in marmoset A1 neurons in single-unit recordings [5,7] and Ca^2+^ imaging [8]. However, most of these neurons also respond to the basic temporal and spectral structures of CVs, such as the temporal modulation frequency in Twitter sounds (8–10 Hz) and the spectral frequency in Phee calls (8–10 kHz) [5,7]. These results suggest that A1 mainly encodes acoustic features carried by conspecific calls rather than biological meanings of vocalizations. In contrast to the findings in A1, our results demonstrate that neurons in the amygdala are specifically activated by marmoset calls, but show little response to calls from other species, natural and artificial sounds, and laboratory-composed acoustic stimuli (Figs 1–3). These results indicate that call-responsive neurons in the amygdala encode aspects of marmoset vocalizations beyond acoustic features. As we know, a marmoset can produce different calls in different social contexts or behavioral conditions. For example, marmosets emit more Phees in an antiphonal calling scenario and during social communication with conspecifics in a wild forest. They produce Trill when they are spatially closed, and emit the food call Chirp when they eat. Therefore, some combinations of spectrotemporal acoustic features in vocalizations may be associated with certain behavioral contexts, emotions and biological meanings, either innately, during development, or through experience. So, different marmoset calls may carry different behavioral, environmental and emotional information, which may activate different amygdala neurons. Call-responsive neurons have also been observed in the amygdala of rodents and big brown bats [24–31] in the last decade, although their calls were used in specific scenarios, such as male mice during mating, a pup separated from its mother (mice and rats), and echolocation in bats [48]. These studies found that the amygdala has both higher-responsive and lower-responsive neurons, which may be correlated with the difference in social conditions or sub-structures of the amygdala. Therefore, the amygdala might be a higher-level or specialized region for processing the biological meaning of CVs in different species.

Previous studies have shown that call-responsive neurons in the A1 rarely respond to time-reversed CVs [5,7], which is thought to be a remarkable feature for identifying CVs. Interestingly, our results demonstrate that call-responsive neurons in the amygdala either respond to natural calls, a time-reversed repertoire, or both (Fig. 4), and both to simple and compound calls (Trillphee), suggesting that neural response to time-reversed CVs is heterogeneous in the amygdala. The fact that some amygdala neurons only respond to time-reversed or natural calls may increase the contrast between natural and time-reversed calls. However, marmoset calls are usually monosyllabic and display spectral and temporal acoustic structures similar to time-reversed calls, which may carry similar behavioral meanings. Moreover, it remains unknown whether marmosets need to distinguish natural from time-reversed calls in their normal environment. Similar coding mechanisms have been found in the visual system where neurons respond to both normal and inverted faces or faces with the eyes or nose upside down [49]. Although further study is required to test more call exemplars, the different coding schemes of call-responsive neurons in the A1 and amygdala in response to natural and time-reversed calls suggest that the A1 may encode the acoustic features of CVs, whereas the amygdala functions to abstract the biological meaning of CVs.

Human speech is crucial for social communication, largely depending on auditory inputs and neural circuity processing. Human beings and non-human primates can recognize the embedded biological meaning or emotional information in human speech [13] and animal vocalizations [13–16]. Marmosets are arboreal, living in dense forests, which leads to reliance on vocal communication rather than visual signals in certain conditions. Calls between them are the most important method for social interaction and identifying partners in certain conditions [15,50], especially Phee calls for caller [51] or gender [34] identity. However, the neural mechanism in the auditory system for recognizing different Phee calls is unclear. Interestingly, we found that different neurons in the amygdala displayed heterogeneous activation by different Phees that possess similar spectral and temporal acoustic features. Strikingly, some neurons in the amygdala showed different responses to different Phee calls from different animals (Fig. S6). Also, Phee integrity is essential for the activation of amygdala Phee-responsive neurons (Fig. 5). These results inspire us to speculate that Phee-responsive neurons in the amygdala may be important for social context-related stimuli or identity discrimination in the auditory modality.

Similar to processing in the face patch system, CVs are processed and perceived by a series of discrete, interconnected cortical areas in non-human primates: the A1 [5], ST [52], insular cortex [53] and ventrolateral prefrontal cortex (vlPFC) [19]. The A1 is surrounded by the temporal lobe, which encodes and integrates the spectral and temporal acoustic information embedded in calls and displays a response preference for CVs [5]. Most call-responsive neurons in the caudal insular cortex do not represent the temporal and spectral structure of a stimulus but respond with long-lasting, sustained firing in response to multiple CVs [53], suggesting that the insular cortex encodes CVs beyond their spectral and temporal acoustic parameters, but shows less selectivity when it comes to CVs [53]. Most vlPFC neurons prefer multiple calls and are not based on the function and meaning of the call [19]. Different from those brain regions, our results indicate that most amygdala neurons selectively respond to one or two species-specific calls and are not driven by other acoustic similar sounds. These results suggest that the amygdala is the most responsive region for CVs, indicating that call-responsive neurons in the amygdala encode the behavioral or emotional information of a call. This speculation is supported by the different responses to Phees from different marmosets (Fig. S6) and similar responses for both natural and time-reversed CVs (Fig. 4). However, further studies are required to explore whether call-responsive neurons in the amygdala encode the behavioral meanings of CVs in a freely moving behavioral condition. Because the amygdala has been implicated in the processing of emotionally relevant visual [21,54,55] and vocal stimuli [21,56], it is possible that call-responsive neurons in the amygdala encode the emotional valence carried by CVs. However, we found that the majority of amygdalar neurons only responded to a specific type of call, and it is not possible to categorize these CVs into neutral, positive or negative valence. Besides, different conspecific calls may carry different emotional information, which inspires us to think that the amygdala may process the emotional valence carried in CVs in a complex manner, such as excited, satisfied, sad, scary or angry, but not in a simple neutral, positive or negative manner.

The macaque is one of the most crucial non-human primate models when it comes to exploring the function of the amygdala in social communication and affective sensory processes. And vision has dominated the spectrum of stimuli for a long time [22,23,57]. However, the macaque only uses 4–5 calls during social interaction and only a few studies have addressed social vocal processes in the amygdala in non-human primates [17,22]. Previous studies have indicated that marmosets and humans have superior skills to macaques in discriminating vocalizations produced by conspecifics [32]. Also, we found that the neural responses elicited by marmoset calls in the amygdala reproduced, in detail, what has been shown not only for vocalizations in other animal models [24–31] but also for face identity, facial expressions and gaze direction in the macaque amygdala [22,57]. These similarities include, but are not restricted to, the relative proportion of stimulus-responsive neurons, the lack of anatomical clustering of neurons with similar response profiles, the slow response latencies and importantly the sharp tuning of these neurons to unique complex sounds. Thus, the marmoset, macaque and human amygdala play similar roles in discriminating socially relevant stimuli, independent of sensory modality.

There are several limitations in the present study. First, we only used seven exemplars of vocalizations which may be insufficient to study the embedded social and emotional information in CVs. Second, the selection of calls from other animal species (dog, bird and tiger) and natural sounds (water flow and bell) was not well acoustically balanced when compared to the marmoset calls. All five sounds have a frequency that is higher in power below 3 kHz, which is different from marmoset calls. Third, although we found that amygdala neurons showed different activation to Phee calls from different callers, only three Phees from two callers were used in the current study, which is not sufficient to address the question of encoding specificity for vocal identity. Further study is required to study the role of the amygdala in encoding the emotional valence and individual identity embedded in conspecific calls, i.e. by presenting more call types from different callers, as well as considering the different social relationships between the listeners and callers. Last, we did not record call-evoked activity from A1 by using the same stimulus set, and thus lack a direct comparison between A1 and the amygdala, which could also be performed in the future. Taken together, despite the limitations of this study, we found that the amygdala is a novel specialized brain region for CV processing and may be a link between auditory cortical regions and higher-order cognitive regions for CV perception.

## MATERIALS AND METHODS

Detailed materials and methods are available in the Supplementary Data.

## Supplementary Material

nwad194_Supplemental_Files
