# Supplementary material for: Representation of conspecific vocalizations in amygdala of awake marmosets
Source: Natl Sci Rev. 2023 Jul 13;10(11):nwad194. doi: 10.1093/nsr/nwad194 (PMC10561708; doi:10.1093/nsr/nwad194)
Supplement: nwad194_Supplemental_Files [file nwad194_supplemental_files.zip › suppmentary figure legends.docx]

**Figs. S1 to S6**

**Fig. S1 Two example neurons showing selective responses to chatter and trill calls.**

(A) Spectrograms of Phee (a pure tone-like long-duration call), Twitter (a string of short upward sweep call), Cry (a broadband call), Chatter, Trill, Chirp, and Tsik calls. (B) Two examples showing selective neural responses to chatter (upper, Student’s t-test, p<0.01) and trill calls (lower, Student’s t-test, p<0.001). Red horizontal dashed line: the averaged spontaneous firing rate averaged over the period before the onset of vocal stimuli across 8 trials. Green shading, periods of acoustic stimulation. (C) Correlation between selectivity index and spontaneous rate.

**Fig. S2 Percentage of CV neurons for each call type in two subjects respectively.**

(A, B) Percentage of CV neurons for each call type in subject F5(**A**) and subject AE(**B**). (C, D) Proportions of amygdala CV neurons in response to 1, 2, and >2 calls in subject F5(**C**) and subject AE(**D**).

**Fig. S3 Temporal and spectra structures of other complex sounds and example neurons responding to natural sound, other species call and lab-composed stimuli.**

(A) Upper, vocal sounds from dog, tiger, and bird; lower, sounds of water flow and bell. (B) Example neurons in response to natural sounds. (C) Example neurons in response to other species’ calls. (D) Example neuron in response to pure tones with different frequencies. (E) Example neuron in response to white noise at different SPL.

**Fig. S4 Rescaled figures for an example Phee-selective neuron in response to pure tone (A), white noise (B) and sAM tone (C) as shown in Fig. 3B-D.**

**Fig. S5 Percentage of Trillphee-selective neurons in response to natural and time-reversed calls.**

**Fig. S6 Amygdala neurons responded differently to different Phees.**

(A) Spectrograms of three phee calls produced by two marmosets (M and X). **Three Phees have different durations from 0.8s-1.8s.** (B-F) Raster (upper), and firing-rates (lower) of five example neurons in response to the 3 phee calls in **a (**one-way ANOVA followed by Student’s t-test, F=30.78, 5.5, 0.01, 30.79,10.44 respectively.). *, p<0.05, **, p<0.01, ***, p<0.001. (E) Example neuron only responding to phee from marmoset M. (F) Example neuron only responding to phee from marmoset X. (G) Percentages of neurons (total 47 neurons) responding to 1, 2, or 3 phees. (H) Distribution of selective index of phee neurons. (I) 47% of amygdala neurons show distinct responses to phee calls from different callers.

**Tables 1-5**

Table 1. Description of measured acoustic features from different calls and call types.

| Name | Description |
| --- | --- |
| F_dom_ (kHz) | Frequency corresponding to the maximum in the spectrum |
| F_max_ (kHz) | Maximum frequency within a call |
| F_min_ (kHz) | Minimum frequency within a call |
| T_fdom_ (s) | The time onset of the dominant frequency within a call |
| F_start_ (kHz) | Starting frequency within a call |
| F_end_ (kHz) | Ending frequency within a call |
| F_bw_ (kHz) | Frequency bandwidth across a call |
| Avg Entropy (dB) | Averaged entropy across a call |
| Dur (s) | Length of a call |

Table 2. Spectrotemporal features of marmoset calls in our acoustic stimulation pool

| Feature | Phee | Twitter | Cry | Chatter | Trill | Chirp | Tsik |
| --- | --- | --- | --- | --- | --- | --- | --- |
| F_dom_ (kHz) | 7.58 | 8.91 | 7.03 | 2.44 | 8.25 | 3.09 | 13.41 |
| F_max_ (kHz) | 8.37 | 11.85 | 13.99 | 4.19 | 9.56 | 4.48 | 13.79 |
| F_min_ (kHz) | 5.92 | 5.64 | 4.18 | 1.15 | 6.99 | 1.73 | 1.56 |
| T_fdom_ (s) | 0.56 | 1.28 | 0.68 | 0.79 | 0.09 | 0.86 | 0.05 |
| F_start_ (kHz) | 6.37 | 7.36 | 5.90 | 2.13 | 8.41 | 3.18 | 12.74 |
| F_end_ (kHz) | 6.95 | 8.21 | 6.63 | 2.22 | 7.24 | 2.26 | 5.01 |
| F_bw_ (kHz) | 2.45 | 6.21 | 9.81 | 3.04 | 2.57 | 2.75 | 12.23 |
| Avg Entropy (dB) | 1.76 | 4.06 | 4.63 | 3.67 | 1.80 | 2.41 | 5.27 |
| Dur (s) | 0.92 | 1.61 | 0.83 | 1.09 | 0.57 | 1.67 | 0.37 |

Table 3. Spectrotemporal features of natural and time-reversed calls

| Feature | Phee M | Phee X1 | Phee X2 | Twitter | Cry | Chatter | Trill | Chirp | Tsik | Trillphee |
| --- | --- | --- | --- | --- | --- | --- | --- | --- | --- | --- |
| F_dom_ (kHz) | 7.58 | 7.50 | 7.26 | 8.91 | 7.03 | 2.44 | 8.25 | 3.09 | 13.41 | 7.97 |
|  | 7.58 | 7.50 | 7.26 | 8.91 | 7.03 | 2.44 | 8.25 | 3.09 | 13.41 | 7.97 |
| F_max_ (kHz) | 8.37 | 7.84 | 7.76 | 11.85 | 13.99 | 4.19 | 9.56 | 4.48 | 13.79 | 9.98 |
|  | 8.37 | 7.84 | 7.76 | 11.85 | 13.99 | 4.19 | 9.56 | 4.48 | 13.79 | 9.98 |
| F_min_ (kHz) | 5.92 | 6.73 | 6.56 | 5.64 | 4.18 | 1.15 | 6.99 | 1.73 | 1.56 | 7.07 |
|  | 5.92 | 6.73 | 6.56 | 5.64 | 4.18 | 1.15 | 6.99 | 1.73 | 1.56 | 7.07 |
| T_fdom_ (s) | 0.56 | 0.37 | 0.45 | 1.28 | 0.68 | 0.79 | 0.09 | 0.86 | 0.05 | 0.21 |
|  | 0.34 | 1.13 | 1.35 | 0.33 | 0.15 | 0.30 | 0.48 | 0.81 | 0.32 | 0.94 |
| F_start_ (kHz) | 6.37 | 6.73 | 6.56 | 7.36 | 5.90 | 2.13 | 8.41 | 3.18 | 12.74 | 7.72 |
|  | 6.95 | 7.84 | 7.76 | 8.21 | 6.63 | 2.22 | 7.24 | 2.26 | 5.01 | 9.84 |
| F_end_ (kHz) | 6.95 | 7.84 | 7.76 | 8.21 | 6.63 | 2.22 | 7.24 | 2.26 | 5.01 | 9.84 |
|  | 6.37 | 6.73 | 6.56 | 7.36 | 5.90 | 2.13 | 8.41 | 3.18 | 12.74 | 7.72 |
| F_bw_ (kHz) | 2.45 | 1.11 | 1.20 | 6.21 | 9.81 | 3.04 | 2.57 | 2.75 | 12.23 | 2.91 |
|  | 2.45 | 1.11 | 1.20 | 6.21 | 9.81 | 3.04 | 2.57 | 2.75 | 12.23 | 2.91 |
| Avg Entropy (dB) | 1.76 | 1.45 | 1.34 | 4.06 | 4.63 | 3.67 | 1.80 | 2.41 | 5.27 | 2.15 |
|  | 1.76 | 1.45 | 1.34 | 4.06 | 4.63 | 3.67 | 1.80 | 2.41 | 5.27 | 2.15 |
| Dur (s) | 0.92 | 1.50 | 1.78 | 1.61 | 0.83 | 1.09 | 0.57 | 1.67 | 0.37 | 1.15 |
|  | 0.92 | 1.50 | 1.78 | 1.61 | 0.83 | 1.09 | 0.57 | 1.67 | 0.37 | 1.15 |

Footnote: light blue indicates natural call while white indicates reversed call.

Table 4. Spectrotemporal features of three different Phee calls from two callers.

| Feature | Phee M | Phee X1 | Phee X2 |
| --- | --- | --- | --- |
| F_dom_ (kHz) | 7.58 | 7.50 | 7.26 |
| F_max_ (kHz) | 8.37 | 7.84 | 7.76 |
| F_min_ (kHz) | 5.92 | 6.73 | 6.56 |
| T_fdom_ (s) | 0.56 | 0.37 | 0.45 |
| F_start_ (kHz) | 6.37 | 6.73 | 6.56 |
| F_end_ (kHz) | 6.95 | 7.84 | 7.76 |
| F_bw_ (kHz) | 2.45 | 1.11 | 1.20 |
| Avg Entropy (dB) | 1.76 | 1.45 | 1.34 |
| Dur (s) | 0.92 | 1.50 | 1.78 |

Table 5. Spectrotemporal features of intact and segmented Phees.

| Feature | Phee | ½ switch | Last 2/3 | First 2/3 |
| --- | --- | --- | --- | --- |
| F_dom_ (kHz) | 7.26 | 7.26 | 6.67 | 7.26 |
| F_max_ (kHz) | 7.76 | 7.76 | 7.76 | 7.39 |
| F_min_ (kHz) | 6.56 | 6.56 | 6.63 | 6.56 |
| T_fdom_ (s) | 0.45 | 1.35 | 0.09 | 0.45 |
| F_start_ (kHz) | 6.56 | 7.09 | 6.63 | 6.56 |
| F_end_ (kHz) | 7.76 | 7.09 | 7.76 | 7.39 |
| F_bw_ (kHz) | 1.20 | 1.20 | 1.13 | 0.83 |
| Avg Entropy (dB) | 1.34 | 1.34 | 1.33 | 1.27 |
