# Supplementary material for: Representation of conspecific vocalizations in amygdala of awake marmosets
Source: Natl Sci Rev. 2023 Jul 13;10(11):nwad194. doi: 10.1093/nsr/nwad194 (PMC10561708; doi:10.1093/nsr/nwad194)
Supplement: nwad194_Supplemental_Files [file nwad194_supplemental_files.zip › suppmentary materials and method.docx]

**Supplementary Materials and Methods**

All experimental procedures were approved by Animal Use and Care Committee of Zhejiang University and were in compliance with the NIH guidelines.

**Animal preparation**

Experiments were conducted on one male and one female adult (2 years old) common marmoset (*Callithrix jacchus*). In our marmoset colony, one breeding male, one breeding female, and their offspring live together in a family cage. More than 8 families are housed in one large room. The experimental animals for single-unit recordings in the amygdala were housed in the same room after head-cap implantation surgery. The conspecific calls used in this study were recorded in a double-wall sound-proof room and were produced by marmosets from the same colony. So, the listeners were familiar with the callers, but not a breeding pair, and not a family member, either. Breeders take care of the animals at least 3 times per day. As the marmoset is a highly vocal non-human primate, each animal is exposed to conspecific calls and human speech every day. However, all marmosets are laboratory-raised animals and they have little chance to be exposed to other types of sounds such as pure tones, natural sounds, and vocalizations from other species.

The animals were prepared based on the chronic awake preparation as described in our previous study [1-5]. In brief, the animals were trained to sit quietly for ~2 h every day in a customized primate chair for ~2 weeks. head-cap implantation surgery was performed under aseptic conditions, during which two posts were attached to the skull to fix the head during electrophysiological recordings. Two dental cement recording chambers were attached over the temporal lobes in both hemispheres and the lateral sulcus was traced as a landmark for locating the auditory cortex [2-4] and amygdala.

**Electrophysiological recordings**

Electrophysiological recordings were based on our previous studies [2-4] and were performed using high-impedance tungsten microelectrodes (2–5 MΩ, FHC Inc., Bowdoin, ME). The microelectrodes were controlled by a one-axis motorized stereotaxic micromanipulator (DMA1510, Narishige). The acoustic stimuli were delivered by a combined workstation composed of TDT S3 modules (TDT Corp., USA) and AlphaLab SNR modules (Alpha Omega Engineering, Israel). The neural signals were amplified (AlphaLab SNR, Alpha Omega Engineering) and digitized (RX6, Tucker-Davis Technologies), then analyzed and saved using custom programs written in MatLab (MathWorks, USA). Spikes were detected online using a template-matching method (AlphaLab SNR, Alpha Omega Engineering) [2-4].

Because our animals were previously used in recording from the auditory cortex (to study temporal processing in the primary auditory cortex by playing time-varying stimuli) and the top of their heads were covered by a thick concrete head-cap, the amygdala was reached from two dental cement chambers above the temporal lobe. Based on the marmoset brain atlas and magnetic resonance imaging (MRI), the amygdala can be reached from the inferior region of the rostral lateral sulcus. To do this, a 1 mm diameter hole was made and the dura was kept intact. A tungsten electrode was then penetrated through the dura at a 50° angle using a microelectrode manipulator with a step size of 200 μm. When reaching a depth of 5.5 mm, the electrode was advanced slowly in 20-μm steps and the acoustic stimuli were played simultaneously. The recording depth of the amygdala ranged between 6 and 9 mm. When single-unit spikes were isolated, acoustic stimuli (marmoset calls, pure tones, broad-band white noise, and sinusoidal amplitude modulated sounds) were played through a high-fidelity speaker ~1.2 m in front of the animals. If the neuron responded to a certain call or several calls, then other acoustic stimuli were played and data were saved, otherwise the current neuron was discarded. Marmoset calls were repeated 6–8 times and other stimuli at least 6 times. When each recording session finished, the marmosets were fed yogurt as a reward. A total of 4 recording holes were opened for the unilateral amygdala.

**Acoustic stimuli**

Acoustic stimuli were played based on our previous methods [5]. All recording sessions were carried out in a double-walled, soundproof chamber (Foshanhenqi, Guangzhou, China). Acoustic signals were generated digitally at a sampling rate of 97.7 kHz using custom MATLAB software (MathWorks), low-pass filtered at 48.8 kHz, converted to analog signals (RX6; Tucker-Davis Technologies, Alachua, FL), power amplified (PM5005, Marantz), attenuated with two serially-linked attenuators (PA5, Tucker-Davis Technologies), and delivered in free-field through a speaker (8351A, Genelec) located ~1.2 m in front of the animal’s head [5]. The sound level in different frequencies of the speaker was calibrated between 0.5-32 kHz at 90 dB using our previous methods.

The auditory stimuli were presented to animals based on our previous methods [6]. Each recording trial includes 3 segments which have a window of baseline before acoustic stimulation, acoustic stimulation and post-stimulation. The details are indicated as follows: the marmoset calls varied in duration from 0.4-1.8 s and have 1 s pre-stimulus period and 2 s post-stimulus period. The trial end to start interval was 3 s. The duration of pure tone was 200 ms with 200 ms pre-stimulus period and 300 ms post-stimulus period. The duration of sinusoidal amplitude modulated tone (AM tone) was 500 ms with 500 ms pre-stimulus period and 500 ms post-stimulus period. To further identify the specificity of representation of conspecific calls in the amygdala, we built an acoustic library with more than a hundred stimuli including 7 marmoset calls, natural sounds (water flow), artificial sounds (bell), vocalizations from other species (e.g., dog, tiger, and bird) as well as laboratory-composed stimuli (pure tones, white noise, and sinusoidal amplitude modulation sounds). Because some natural sounds have longer duration, the trial length in this study is in the range of ~6-11 s. In this study, except the white noise during sound level tuning test, all the stimuli were presented to animals at 60 dB. To test the encoding property of amygdala neurons to conspecific vocalization (CV), single-syllables of marmoset calls (Phee, Twitter, Cry, Chatter, Trill, Chirp, and Tsik) recorded from a marmoset colony were presented to animals pseudo-randomly (Tables 1-2). In the colony, the listeners and callers can see and hear each other in their own homecage; however, they were not a breeding pair, or not a family member, either. The listeners were familiar with the callers. Calls from other species (dog, bird, and tiger), the natural sound of water-flow, and an artificial bell sound were obtained from www.freesound.org. Pure tones, broad-band noise, and sinusoidal amplitude modulated sounds were synthetized using MATLAB. Time-reversed CVs (Table 3) and 3 Phees from two marmosets (Table 4) were used to examine the coding of behavioral meaning by amygdala neurons. Last, to test whether Phee-responsive neurons depend on the integrity of Phees, we created partial-removed Phees and half-switch Phees (Table 5). For the half-switch Phee, we cut the Phee calls into halves from the middle and switched the sequence of paired segments, which did not change the energy power and spectrogram of the Phee calls. For the partial-removed Phee, we removed first 1/3 or last 1/3 of Phee calls. This modification would shorten the time and reduce the spectrum structure of the Phee calls.

In present study, the acoustic stimuli were played in the following sequences in three sessions: (1) marmoset calls (7 types) together with other species calls, natural sound, lab-composed sound, pure tone, white noise and sAM tone; (2) 8 types of calls and time-reversed calls; (3) Phee call, time-switched Phee and partially removed Phee. We used the first group calls to determine whether a neuron is a call-responsive neuron. Only call-responsive neurons were tested by all time-reversed CV calls (8 types).

**Data analysis**

Data were analyzed using a customized script written in MatLab. The analysis methods used were the same as in our previous publications [2,4,7,8]. In brief, the driven rate elicited by sound was calculated over the entire stimulus duration in 50-ms bins and the mean spontaneous rate (estimated over the entire stimulus set) was subtracted in all analyses. In this study we found the firing of amygdala neurons could return to baseline. So, the spontaneous rate was calculated in time of 2 s before stimulation. Significant driven activity was defined by the activity difference between the driven rate (dr) and the spontaneous rate (sr) [paired Student’s test, driven rate >2 standard deviations higher than the average spontaneous rate (asr)].

$$dr\geq\left( sr+2*std (sr) \right)$$

dr, average driven rate; sr, average spontaneous firing rate; std, standard deviation of spontaneous firing rate.

In our task, if a neuron shows the highest activity to one call and the averaged driven activity is higher than averaged spontaneous activity plus 2 times the standard deviation of spontaneous activity, the neuron is defined as a call-responsive neuron. We used the same criterion to define the time-reversed neurons. The population activity of 76 amygdala call-responsive neurons in response to different calls were first averaged across trials, then normalized by the maximum activity in Phee, Twitter, and Cry calls. The activity of different neurons was sorted based on maximum activity. Clearly, a large population of neurons were activated by the Phee call, and a few neurons were for Twitter and Cry (Fig. 2A).

To calculate the response latency, data were re-analyzed in 4-ms bins. Only the driven activity in 3 consecutive bins (12 ms) that was significantly higher than spontaneous activity (paired Student’s test) was significant activation by an acoustic stimulus. The first response bin was calculated as the latency of CV-evoked amygdala neurons. The latency of several neurons >250 ms was defined as 250 ms.

The selectivity index (SI) for CVs was calculated based on previous methods [9]. The SI was calculated using the absolute value of the averaged responses to each stimulus minus the baseline firing rate. The SI is a measure of the depth of selectivity across all 7 CVs presented and is defined as:

$$SI=(n-\sum_{i=1}^{n} (\lambda i/\lambda max))/(n-1)$$

where n is the total number of stimuli,$\lambda i$ is the firing rate of the neuron to the ith stimulus and $\lambda max$ is the neuron’s maximum firing rate to one of the stimuli [10]. Thus, if a neuron is responsive and responds to only one stimulus and not to any other stimulus, the SI would be 1. If the neuron responded identically to all stimuli, the SI would be 0.

The SI for natural and time-reversed calls was defined as follows:

$$SI=({dr}_{vocal}-{dr}_{reverse})/({dr}_{vocal}+{dr}_{reverse})$$

${dr}_{vocal}$, driven rate for CV stimuli; ${dr}_{reverse}$, driven rate for time-reversed CVs.

Caller-responsive neurons were calculated as whether the driven rate to a Phee call of marmoset M was higher or lower than that of two marmoset X Phee calls.

All values are expressed as the mean ± sem unless otherwise speciﬁed. Data in the results were analyzed with ANOVA followed by the paired t-test. P values <0.05 were considered statistically significant for all the analyses (*p <0.05, **p <0.01, ***p <0.001).

**Histological staining**

At the cessation of the electrophysiological recordings, small electrolytic lesions were made in physiologically identified regions by passing 30 µA through the recording electrode for 120 s (A365 stimulus isolator, World Precision Instruments, Inc.).

Animals were euthanized by administering an overdose of sodium pentobarbitone (100 mg/kg) and transcardiac perfusion with 0.9% saline followed by 4% paraformaldehyde in 0.1 M phosphate buffer (pH 7.4). After perfusion, the brain was removed, immersed in 30% sucrose solution, and frozen. Sections (30 µm) were cut on a freezing microtome through the entire extent of the amygdala and processed using the Nissl staining method. Last, the recording location was checked with a confocal microscope.

To further identify the location of recorded call-responsive neurons, we first projected the recording locations of 3 hemispheres onto one standard marmoset brain based on fMRI imaging. The electrode depths were first calibrated by staining and histology. Then the insertion path of electrode was plotted based on its angle and depth. Call-responsive neurons are indicated in different colors; non-responsive neurons are indicated in black.

**Reconstruction of recording map**

To reconstruct the recording map of call-responsive neurons in the marmoset amygdala, we first calibrated the depth of recorded units based on the insertion depth of electrodes in the brain and the depth of electrolytic lesion in Nissl-stained section. Then we rotated the recording sites 180º for the right hemisphere. Third, we reconstructed the electrodes’ track and locations of each recorded neuron in the left hemisphere of amygdala. Last, we put the reconstruction of recording map to the normalized marmoset brain based on the marmoset brain atlas (Fig. 2G).

**Reference**

1. Lu, T, Liang, L, Wang, X. Temporal and rate representations of time-varying signals in the auditory cortex of awake primates. *Nat Neurosci*. 2001; **4**(11): 1131-8.

2. Gao, L, Kostlan, K, Wang, Y*, et al.* Distinct Subthreshold Mechanisms Underlying Rate-Coding Principles in Primate Auditory Cortex. *Neuron*. 2016; **91**(4): 905-19.

3. Gao, L, Wang, X. Intracellular neuronal recording in awake nonhuman primates. *Nat Protoc*. 2020; **15**(11): 3615-31.

4. Gao, L, Wang, X. Subthreshold Activity Underlying the Diversity and Selectivity of the Primary Auditory Cortex Studied by Intracellular Recordings in Awake Marmosets. *Cereb Cortex*. 2019; **29**(3): 994-1005.

5. Wang, X, Zhang, Y, Bai, S*, et al.* Corticofugal Modulation of Temporal and Rate Representations in the Inferior Colliculus of the Awake Marmoset. *Cereb Cortex*. 2022. doi: 10.1093/cercor/bhab467.

6. Wang, X, Zhang, Y, Zhu, L*, et al.* Selective corticofugal modulation on sound processing in auditory thalamus of awake marmosets. *Cereb Cortex*. 2022. doi: 10.1093/cercor/bhac278.

7. Lu, T, Liang, L, Wang, X. Neural representations of temporally asymmetric stimuli in the auditory cortex of awake primates. *J Neurophysiol*. 2001; **85**(6): 2364-80.

8. Bendor, D, Wang, X. Neural response properties of primary, rostral, and rostrotemporal core fields in the auditory cortex of marmoset monkeys. *J Neurophysiol*. 2008; **100**(2): 888-906.

9. Plakke, B, Diltz, MD, Romanski, LM. Coding of vocalizations by single neurons in ventrolateral prefrontal cortex. *Hear Res*. 2013; **305**: 135-43.

10. Wirth, S, Avsar, E, Chiu, CC*, et al.* Trial outcome and associative learning signals in the monkey hippocampus. *Neuron*. 2009; **61**(6): 930-40.
