# Supplementary material for: Representation of conspecific vocalizations in amygdala of awake marmosets
Source: Natl Sci Rev. 2023 Jul 13;10(11):nwad194. doi: 10.1093/nsr/nwad194 (PMC10561708; doi:10.1093/nsr/nwad194)
Supplement: nwad194_Supplemental_Files [file nwad194_supplemental_files.zip › suppmentary tables.docx]

**Supplementary Tables 1-5**

Table 1. Description of measured acoustic features from different calls and call types.

| Name | Description |
| --- | --- |
| F_dom_ (kHz) | Frequency corresponding to the maximum in the spectrum |
| F_max_ (kHz) | Maximum frequency within a call |
| F_min_ (kHz) | Minimum frequency within a call |
| T_fdom_ (s) | The time onset of the dominant frequency within a call |
| F_start_ (kHz) | Starting frequency within a call |
| F_end_ (kHz) | Ending frequency within a call |
| F_bw_ (kHz) | Frequency bandwidth across a call |
| Avg Entropy (dB) | Averaged entropy across a call |
| Dur (s) | Length of a call |

Table 2. Spectrotemporal features of marmoset calls in our acoustic stimulation pool

| Feature | Phee | Twitter | Cry | Chatter | Trill | Chirp | Tsik |
| --- | --- | --- | --- | --- | --- | --- | --- |
| F_dom_ (kHz) | 7.58 | 8.91 | 7.03 | 2.44 | 8.25 | 3.09 | 13.41 |
| F_max_ (kHz) | 8.37 | 11.85 | 13.99 | 4.19 | 9.56 | 4.48 | 13.79 |
| F_min_ (kHz) | 5.92 | 5.64 | 4.18 | 1.15 | 6.99 | 1.73 | 1.56 |
| T_fdom_ (s) | 0.56 | 1.28 | 0.68 | 0.79 | 0.09 | 0.86 | 0.05 |
| F_start_ (kHz) | 6.37 | 7.36 | 5.90 | 2.13 | 8.41 | 3.18 | 12.74 |
| F_end_ (kHz) | 6.95 | 8.21 | 6.63 | 2.22 | 7.24 | 2.26 | 5.01 |
| F_bw_ (kHz) | 2.45 | 6.21 | 9.81 | 3.04 | 2.57 | 2.75 | 12.23 |
| Avg Entropy (dB) | 1.76 | 4.06 | 4.63 | 3.67 | 1.80 | 2.41 | 5.27 |
| Dur (s) | 0.92 | 1.61 | 0.83 | 1.09 | 0.57 | 1.67 | 0.37 |

Table 3. Spectrotemporal features of natural and time-reversed calls

| Feature | Phee M | Phee X1 | Phee X2 | Twitter | Cry | Chatter | Trill | Chirp | Tsik | Trillphee |
| --- | --- | --- | --- | --- | --- | --- | --- | --- | --- | --- |
| F_dom_ (kHz) | 7.58 | 7.50 | 7.26 | 8.91 | 7.03 | 2.44 | 8.25 | 3.09 | 13.41 | 7.97 |
|  | 7.58 | 7.50 | 7.26 | 8.91 | 7.03 | 2.44 | 8.25 | 3.09 | 13.41 | 7.97 |
| F_max_ (kHz) | 8.37 | 7.84 | 7.76 | 11.85 | 13.99 | 4.19 | 9.56 | 4.48 | 13.79 | 9.98 |
|  | 8.37 | 7.84 | 7.76 | 11.85 | 13.99 | 4.19 | 9.56 | 4.48 | 13.79 | 9.98 |
| F_min_ (kHz) | 5.92 | 6.73 | 6.56 | 5.64 | 4.18 | 1.15 | 6.99 | 1.73 | 1.56 | 7.07 |
|  | 5.92 | 6.73 | 6.56 | 5.64 | 4.18 | 1.15 | 6.99 | 1.73 | 1.56 | 7.07 |
| T_fdom_ (s) | 0.56 | 0.37 | 0.45 | 1.28 | 0.68 | 0.79 | 0.09 | 0.86 | 0.05 | 0.21 |
|  | 0.34 | 1.13 | 1.35 | 0.33 | 0.15 | 0.30 | 0.48 | 0.81 | 0.32 | 0.94 |
| F_start_ (kHz) | 6.37 | 6.73 | 6.56 | 7.36 | 5.90 | 2.13 | 8.41 | 3.18 | 12.74 | 7.72 |
|  | 6.95 | 7.84 | 7.76 | 8.21 | 6.63 | 2.22 | 7.24 | 2.26 | 5.01 | 9.84 |
| F_end_ (kHz) | 6.95 | 7.84 | 7.76 | 8.21 | 6.63 | 2.22 | 7.24 | 2.26 | 5.01 | 9.84 |
|  | 6.37 | 6.73 | 6.56 | 7.36 | 5.90 | 2.13 | 8.41 | 3.18 | 12.74 | 7.72 |
| F_bw_ (kHz) | 2.45 | 1.11 | 1.20 | 6.21 | 9.81 | 3.04 | 2.57 | 2.75 | 12.23 | 2.91 |
|  | 2.45 | 1.11 | 1.20 | 6.21 | 9.81 | 3.04 | 2.57 | 2.75 | 12.23 | 2.91 |
| Avg Entropy (dB) | 1.76 | 1.45 | 1.34 | 4.06 | 4.63 | 3.67 | 1.80 | 2.41 | 5.27 | 2.15 |
|  | 1.76 | 1.45 | 1.34 | 4.06 | 4.63 | 3.67 | 1.80 | 2.41 | 5.27 | 2.15 |
| Dur (s) | 0.92 | 1.50 | 1.78 | 1.61 | 0.83 | 1.09 | 0.57 | 1.67 | 0.37 | 1.15 |
|  | 0.92 | 1.50 | 1.78 | 1.61 | 0.83 | 1.09 | 0.57 | 1.67 | 0.37 | 1.15 |

Footnote: light blue indicates natural call while white indicates reversed call.

Table 4. Spectrotemporal features of three different Phee calls from two callers.

| Feature | Phee M | Phee X1 | Phee X2 |
| --- | --- | --- | --- |
| F_dom_ (kHz) | 7.58 | 7.50 | 7.26 |
| F_max_ (kHz) | 8.37 | 7.84 | 7.76 |
| F_min_ (kHz) | 5.92 | 6.73 | 6.56 |
| T_fdom_ (s) | 0.56 | 0.37 | 0.45 |
| F_start_ (kHz) | 6.37 | 6.73 | 6.56 |
| F_end_ (kHz) | 6.95 | 7.84 | 7.76 |
| F_bw_ (kHz) | 2.45 | 1.11 | 1.20 |
| Avg Entropy (dB) | 1.76 | 1.45 | 1.34 |
| Dur (s) | 0.92 | 1.50 | 1.78 |

Table 5. Spectrotemporal features of intact and segmented Phees.

| Feature | Phee | ½ switch | Last 2/3 | First 2/3 |
| --- | --- | --- | --- | --- |
| F_dom_ (kHz) | 7.26 | 7.26 | 6.67 | 7.26 |
| F_max_ (kHz) | 7.76 | 7.76 | 7.76 | 7.39 |
| F_min_ (kHz) | 6.56 | 6.56 | 6.63 | 6.56 |
| T_fdom_ (s) | 0.45 | 1.35 | 0.09 | 0.45 |
| F_start_ (kHz) | 6.56 | 7.09 | 6.63 | 6.56 |
| F_end_ (kHz) | 7.76 | 7.09 | 7.76 | 7.39 |
| F_bw_ (kHz) | 1.20 | 1.20 | 1.13 | 0.83 |
| Avg Entropy (dB) | 1.34 | 1.34 | 1.33 | 1.27 |
