# Supplementary figures and images for: Representation of conspecific vocalizations in amygdala of awake marmosets
Source: Natl Sci Rev. 2023 Jul 13;10(11):nwad194. doi: 10.1093/nsr/nwad194 (PMC10561708; doi:10.1093/nsr/nwad194)

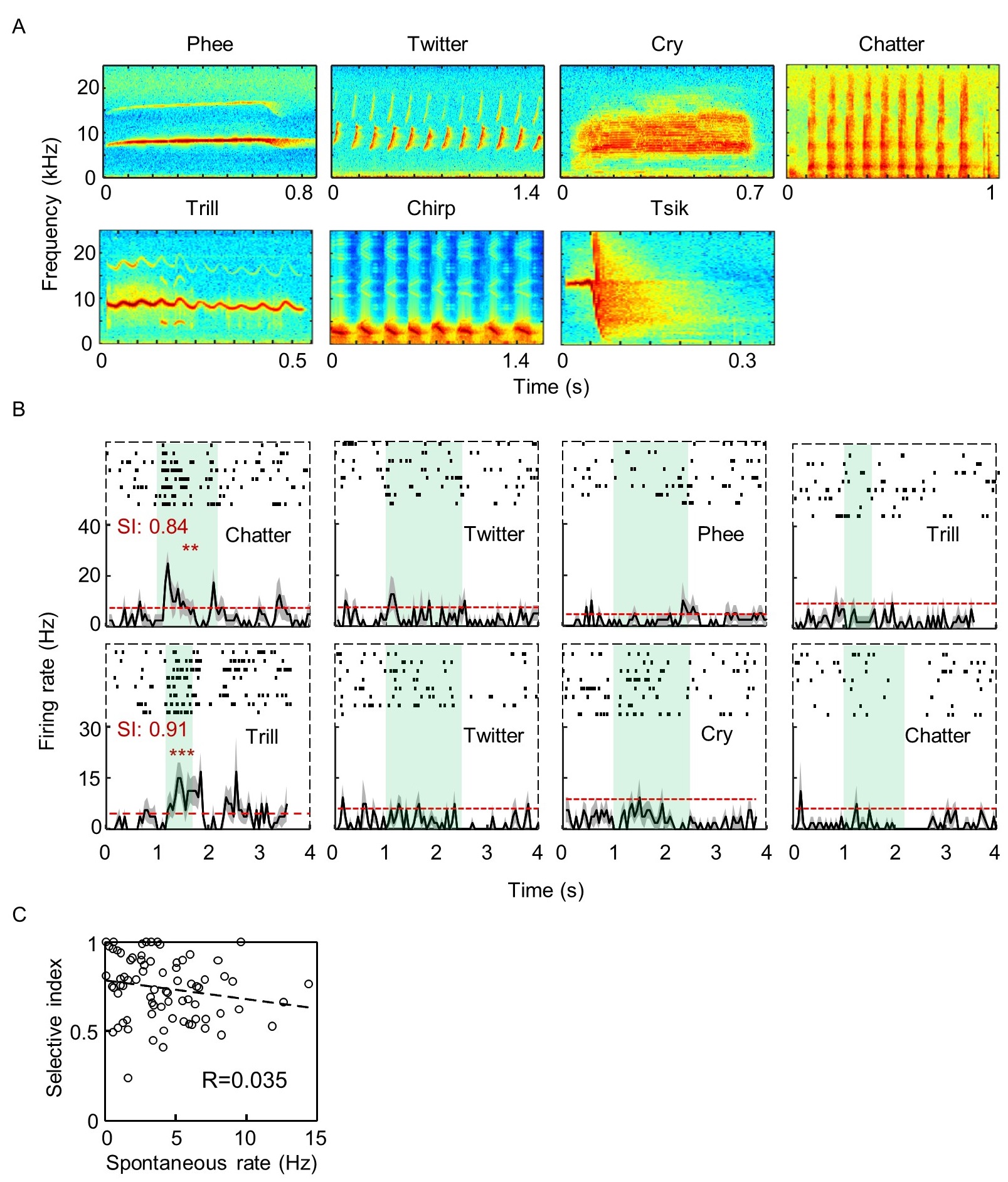

Supplement: nwad194_Supplemental_Files [file nwad194_supplemental_files.zip › S1.jpg]

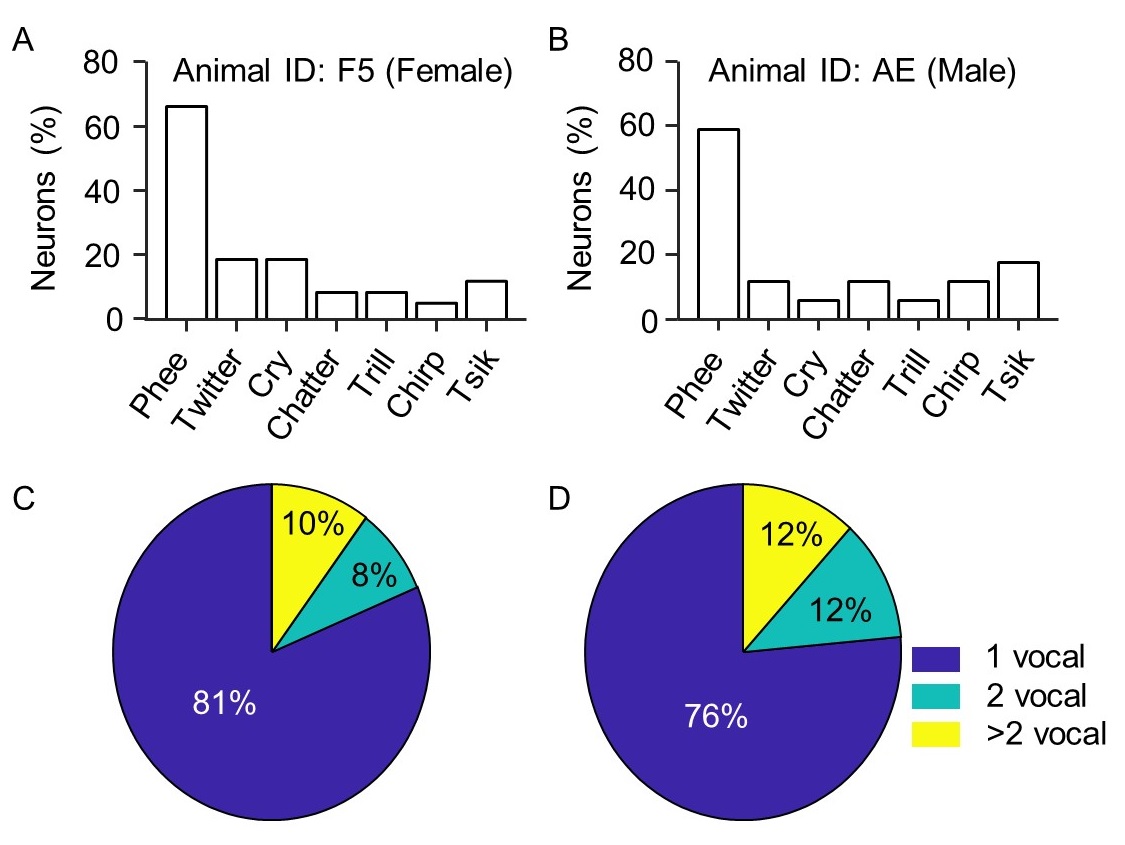

Supplement: nwad194_Supplemental_Files [file nwad194_supplemental_files.zip › S2.jpg]

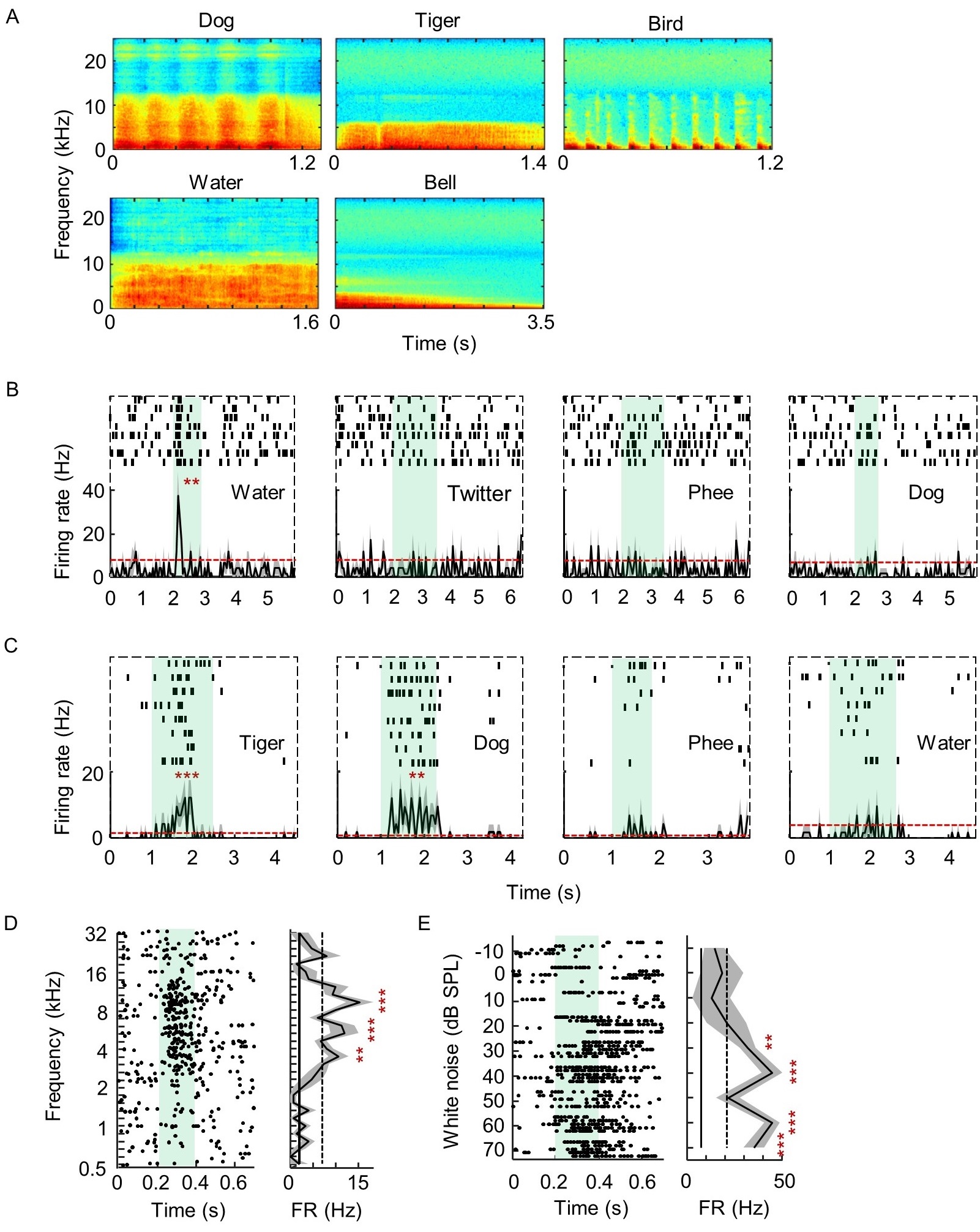

Supplement: nwad194_Supplemental_Files [file nwad194_supplemental_files.zip › S3.jpg]

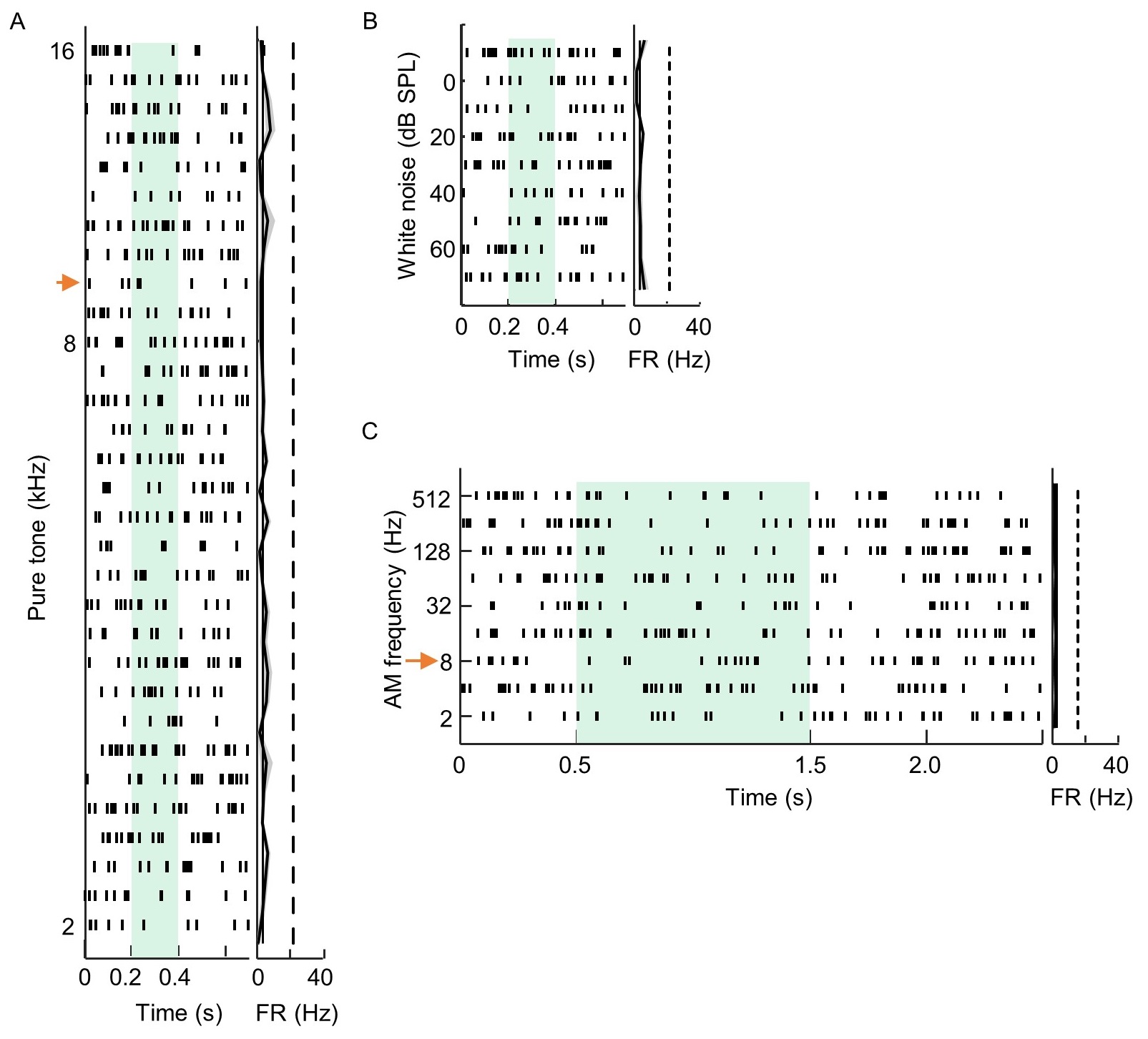

Supplement: nwad194_Supplemental_Files [file nwad194_supplemental_files.zip › S4.jpg]

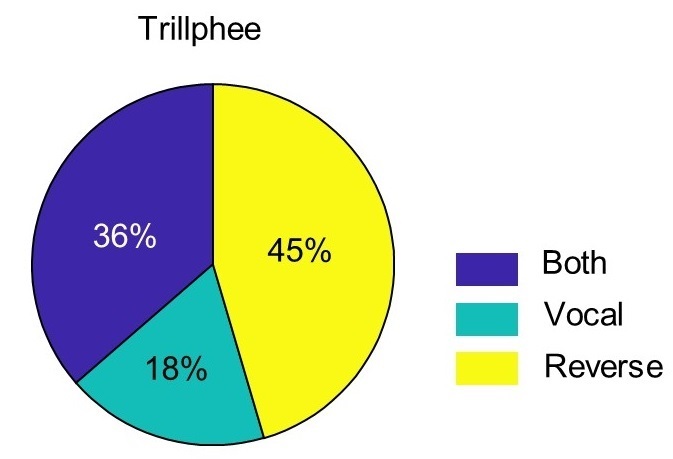

Supplement: nwad194_Supplemental_Files [file nwad194_supplemental_files.zip › S5.jpg]

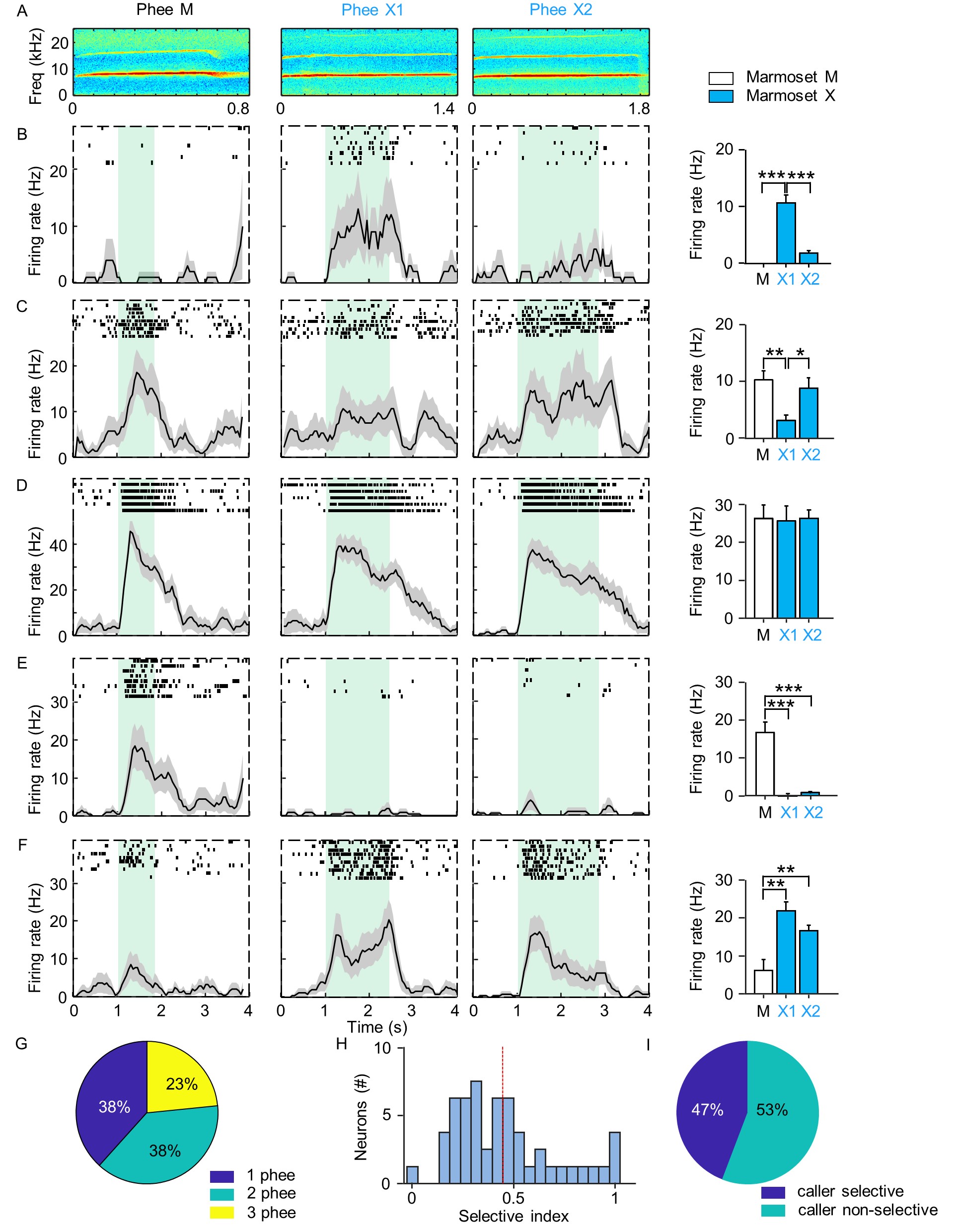

Supplement: nwad194_Supplemental_Files [file nwad194_supplemental_files.zip › S6.jpg]
